# Short-Term Wind Power Prediction Based on Encoder–Decoder Network and Multi-Point Focused Linear Attention Mechanism

**DOI:** 10.3390/s24175501

**Published:** 2024-08-25

**Authors:** Jinlong Mei, Chengqun Wang, Shuyun Luo, Weiqiang Xu, Zhijiang Deng

**Affiliations:** 1 School of Computer Science and Technology, Zhejiang Sci-Tech University, Hangzhou 310018, China; 202230603093@mails.zstu.edu.cn; 2 Key Laboratory of Intelligent Textile and Flexible Interconnection of Zhejiang Province, Zhejiang Sci-Tech University, Hangzhou 310018, China; shuyunluo@zstu.edu.cn (S.L.); wqxu@zstu.edu.cn (W.X.); 3 Fox-Ess, Co., Ltd., Wenzhou 325024, China; dengzhijiang@fox-ess.com

**Keywords:** short-term wind power prediction, encoder–decoder network, LSTM network, multi-point focused linear attention

## Abstract

Wind energy is a clean energy source that is characterised by significant uncertainty. The electricity generated from wind power also exhibits strong unpredictability, which when integrated can have a substantial impact on the security of the power grid. In the context of integrating wind power into the grid, accurate prediction of wind power generation is crucial in order to minimise damage to the grid system. This paper proposes a novel composite model (MLL-MPFLA) that combines a multilayer perceptron (MLP) and an LSTM-based encoder–decoder network for short-term prediction of wind power generation. In this model, the MLP first extracts multidimensional features from wind power data. Subsequently, an LSTM-based encoder-decoder network explores the temporal characteristics of the data in depth, combining multidimensional features and temporal features for effective prediction. During decoding, an improved focused linear attention mechanism called multi-point focused linear attention is employed. This mechanism enhances prediction accuracy by weighting predictions from different subspaces. A comparative analysis against the MLP, LSTM, LSTM–Attention–LSTM, LSTM–Self_Attention–LSTM, and CNN–LSTM–Attention models demonstrates that the proposed MLL-MPFLA model outperforms the others in terms of MAE, RMSE, MAPE, and $R^{2}$, thereby validating its predictive performance.

## 1. Introduction

Wind power is a clean and renewable energy source that is widely used in power systems. As the amount of wind power generation equipment installed increases [1], more wind power is connected to the power grid system. As a renewable natural resource, wind power itself has a high degree of uncertainty, and the amount of generated power is also uncertain, which poses a great safety hazard when connected to the grid. Therefore, the prediction of wind power is an indispensable safety guarantee for power grid security. In general, wind power prediction methods can be divided into three categories: physical methods, statistical methods, and artificial intelligence methods [2]. Physical methods typically utilise wind speed, humidity, pressure, and temperature information from numerical weather prediction (NWP) to model the relationship between wind speed and wind power [3]. The NWP method first predicts the future wind speed, and then calculates the wind power through the wind power curve [4]. However, the NWP method necessitates the utilisation of meteorological prediction products in real time during actual application, which inevitably increases the prediction cost [5]. Statistical methods include autoregressive (AR) models [2], autoregressive moving average (ARMA) models [6], and multiple autoregressive moving average (M-ARMA) models [7]. Because statistical methods make predictions under certain assumptions, this results in most statistical methods being unable to solve the problem of nonlinear time series wind power data prediction [8]. Several scholars have combined statistical methods with machine learning methods to predict wind power data. In the latest wind power prediction research based on the combination of statistical methods and machine learning, Wan et al. [9] proposed a method (CBC) for generating nonparametric prediction distributions using high-order statistics. This method combines machine learning with conditional moments and cumulants, which can describe the overall uncertainty in the prediction process and use the unique additivity of high-order cumulants to quantify the overall uncertainty of the estimated conditional moments. Three different series expansions, namely, Gram–Charlier, Edgeworth, and Cornish–Fisher, were used to improve the overall performance and generalization ability.

With the continuous development of technology, more and more artificial intelligence methods have been proven to have excellent performance in the field of wind power forecasting, including backpropagation neural networks (BP) [10], support vector machines (SVM) [11], and graph neural networks (GNN) [12]. In terms of short-term wind power prediction methods, multilayer perceptrons (MLP), light gradient boosting machines (LightGBM) [13], and convolutional neural networks (CNN) [14] are widely used. Liu et al. [15] proposed a wind farm cluster power prediction model based on power fluctuation pattern recognition and spatiotemporal graph neural network prediction. In this study, the extreme points of the data were first statistically analyzed and the wind farm cluster power was divided into different fluctuation processes. Then, four indicators for judging the division of power fluctuation patterns were summarized from the two aspects of time stability and amplitude fluctuation in these fluctuation processes. Finally, the dynamic spatiotemporal correlation between adjacent wind farm sites was considered under different fluctuation modes and a spatiotemporal graph neural network was used to predict each fluctuation mode. In the latest study on wind power forecasting using graph neural networks, Yang et al. [16] considered the correlation between multiple wind farms and proposed the wind farm cluster (WFC) short-term power forecasting method based on global information adaptive perceptual graph convolution. First, a method for calculating the dynamic correlation coefficient between wind farms was proposed, with the graph structure at each moment obtained through this method. Then, the key features and dynamic correlation coefficients between multiple wind farms were obtained by using graph embedding and clustering algorithms. Finally, an adaptive graph convolution network was established to predict wind power.

Because wind power data represent a kind of time series data, each element has strong temporal correlation. This characteristic of wind power data poses a challenge to the above methods, as they cannot fully capture this relationship. To address this issue, recursive neural network (RNN) [17] approaches have garnered significant interest from scholars. Notable RNN networks, such as long short-term memory (LSTM) neural networks, have demonstrated remarkable efficacy in wind power prediction. Wen et al. [18] proposed a new time series prediction model, LSTM–Attention–LSTM, for nonstationary multivariate time series data. Their model uses two LSTM networks for the encoder and decoder, with an attention mechanism placed between the encoder and decoder. They verified this model based on multiple real datasets, proving that the model can effectively improve the accuracy of multivariate and multistep time series data prediction. Zhou et al. [19] employed the K-means clustering method to categorize diverse factors influencing wind power, and proposed a novel K-means–LSTM prediction model for wind power prediction. Chen et al. [20] conducted a feature screening process on the multiple factors affecting wind power and subsequently proposed a novel wind power prediction model combining CNN and BiLSTM. Tang et al. [21] considered the impact of four meteorological variables on wind power generation: wind speed, wind direction, air pressure, and temperature. They used the CNN–LSTM architecture to extract key feature information from the data and used the attention mechanism to assign different weights highlighting the most critical features, thereby achieving more accurate wind power prediction. Ye et al. [22] divided NWP data according to fluctuation trends, extracted different fluctuation features, and used the improved grey wolf optimizer to optimize the hyperparameters of the LSTM-based Seq2Seq model for prediction. Wang et al. [23] proposed a method for predicting wind power generation through the wind power conversion relationship. In their study, wind speed data were first decomposed into multiple subcomponents using empirical mode decomposition (EMD), then these subcomponents were divided into three frequency components (high, medium, and low frequency) using K-means clustering. Finally, three machine learning models, namely, SVM, XGBoost regression, and Lasso regression, were used to predict these three components. The WPC model was then used to calculate the output power of wind power generation based on the predicted wind speed value. Dai et al. [24] proposed an offshore wind power prediction model based on ensemble empirical mode decomposition (EEMD) and an LSTM network. The input wind power data were decomposed into different signal components using EEMD, while the LSTM network was used to obtain different predicted wind power for each group of decomposed components. These predictions were then combined to obtain the final prediction results. In the latest study on wind power generation prediction based on variable modal decomposition (VMD), Tan et al. [25] used the VMD algorithm to decompose wind power data into several subsequences in order to reduce the nonstationarity of the data, then used BiLSTM for wind power prediction, with an improved MPA method (IMAP) used to optimize the parameters of the BiLSTM network. Lei et al. [26] proposed a soft measurement model based on an LSTM network; they used VMD to preprocess the data and the isolation forest algorithm to detect anomalies in the original sequence during preprocessing. Then, an LSTM network was used to predict each modal component separately and the prediction of each component was summed up and output to obtain better prediction results. Zhong et al. [27] employed principal component analysis to reduce the dimensionality and denoise NWP data, after which they used an LSTM network with hyperparameters optimized by a genetic algorithm (GA) to predict wind power. Zhao et al. [28] utilized a graph convolutional neural network to extract features based on the shared spatial characteristics between wind power data. Subsequently, an LSTM network was employed to extract temporal features and perform wind power prediction based on spatial and temporal characteristics. The above studies demonstrate that artificial intelligence methods are both efficient and feasible for wind power prediction. In particular, recurrent neural networks (RNNs), represented by LSTM networks, are more accurate in capturing temporal correlations and have better prediction performance than traditional shallow networks when applied to predicting time series data such as wind power data.

In order to predict future short-term power generation through NWP data, this paper proposes a novel hybrid prediction model named MLL-MPFLA. The model first employs a multilayer perceptron (MLP) to extract multidimensional features from the wind power dataset, accelerating the feature extraction process. Next, an LSTM-based encoder–decoder model is utilized to capture temporal features within the dataset. The final wind power prediction results are obtained by integrating both the multidimensional and temporal features. Additionally, a multi-point focused linear attention mechanism is introduced into the decoding process of the LSTM-based encoder–decoder model. This approach allows for the weighted combination of different subspace features, enabling comprehensive integration of features across multiple dimensions for more accurate predictions. The main contributions of this paper are as follows:

1. Comprehensive feature extraction: The proposed MLL-MPFLA model combines MLP and an LSTM-based encoder–decoder network to extract both multidimensional and temporal features from the input data. This approach fully considers the influence of multiple variables and temporal correlations on wind power prediction results. Compared to a standalone MLP model, the proposed model takes into account the impact of time series characteristics on power generation. In contrast to the traditional LSTM-based encoder–decoder model, it incorporates multivariate data features. Consequently, the input time series data undergo more extensive feature extraction, leading to improved prediction accuracy.
2. Convolution strategy for time series data: In traditional focused linear attention mechanisms, the deep convolution module may lose some original time series data features during the linear computation of query vectors. To address this problem, in this paper we directly perform convolution operations on the data of each time step to obtain the corresponding data features, thereby replacing the deep convolution module. Direct convolution of the input time step data facilitates more precise and comprehensive feature extraction, particularly for time series data, ultimately enhancing the accuracy of the attention mechanism’s output.
3. Introduction of a multi-point focused linear attention mechanism: Traditional focused linear attention mechanisms often overemphasize features in a specific subspace, potentially leading to insufficient analysis of multidimensional features. Considering the varying characteristics of time series data across different subspaces, this paper proposes a multi-point focused linear attention mechanism. This mechanism employs an improved focused attention mechanism to calculate attention outputs across multiple subspaces, concatenates the outputs from different subspaces, then performs feature projection to generate new outputs. By integrating multidimensional time series features across multiple subspaces, the model more comprehensively captures data characteristics, leading to more accurate predictions. Compared to traditional focused linear attention mechanisms, this improved approach better represents the time series features across multiple subspaces, thereby enhancing prediction performance.
4. Experimental validation: To verify the effectiveness of the proposed model, we conducted comparative experiments using real wind power generation data from a wind farm in Xinjiang, China. The model was compared with the MLP, LSTM, LSTM–Attention–LSTM, LSTM–Self_Attention–LSTM, and CNN–LSTM–Attention models, focusing on three key aspects: performance metrics, error analysis, and prediction effectiveness.

The remainder of this paper is organised as follows: Section 2 provides a concise overview of the pertinent methodologies; Section 3 delineates the overarching model architecture and improvements to the focused linear attention mechanism; Section 4 illustrates the predictive efficacy of the proposed MLL-MPFLA model on wind power data and analyses the experimental outcomes; finally, Section 5 offers a summary and conclusion to the paper.

## 2. Methods and Models

### 2.1. Multilayer Perceptron (MLP)

MLP is a deep learning model based on a feedforward neural network. It can be used to solve various machine learning problems, including classification, regression, and clustering. Additionally, it can be used for data feature extraction [29]. Its structure can be divided into three layers: the input layer, hidden layer and output layer. The input layer and output layer have one layer each, while the hidden layer can have multiple layers. Each node layer is composed of numerous neurons, all of which are fully connected to the previous layer [30]. Each node layer receives the output of the previous layer, performs a nonlinear activation function operation, and obtains the output of the current layer. The input data are received by the input layer of the MLP, processed by the nonlinear activation function of the hidden layer, and finally the processed data are output at the output layer. This hierarchical structure endows MLP with considerable expressive capacity, enabling it to address nonlinear problems and high-dimensional data [31]. In addition, it can be trained using a backpropagation algorithm; following repeated iterations of training, MLP learns the intricate nonlinear relationships between input features, thereby facilitating the extraction of features from the data.

### 2.2. Long Short-Term Memory Neural Network (LSTM)

An RNN is a neural network structure with recurrent connections and that has been specifically designed to process sequence data with time correlations. In an RNN, the connections between the neurons form a loop path, which allows the network to process sequence data step-by-step while retaining the previous information state. Although RNNs have strong expressive power in processing sequence data, they also have several limitations. These include difficulty in capturing the time correlation between long sequence data, gradient vanishing, and gradient explosion. In order to address these issues, Hochreiter and Schmidhuber proposed the LSTM network, which captures long-term dependencies between data by introducing a gating mechanism. LSTM networks have three key gating units and two key variables [32]. The gating units are the input gate, forget gate, and output gate. Among the two key variables, one is primarily responsible for short-term memory, that is, the hidden state *h*, which is used to record the current time step information, while the other is responsible for long-term memory, that is, the cell state C, which is used to record the characteristics of the entire time series data. When time series data pass through these gated units, the hidden state *h* and cell state C are continuously updated and forgotten through learning in order to obtain more accurate dependencies between the data. This process can be represented by the following function [33,34]:

(1) $$I_{t}=\sigma \left(W_{t}\left[x_{t},h_{t-1}\right]+\xi_{i}\right),$$

(2) $$F_{t}=\sigma \left(W_{f}\left[x_{t},h_{t-1}\right]+\xi_{f}\right),$$

(3) $$O_{t}=\sigma \left(W_{o}\left[x_{t},h_{t-1}\right]+\xi_{o}\right),$$

(4) $$\tilde{C_{t}}=tanh\left(W_{c}\left[x_{t},h_{t-1}\right]+\xi_{c}\right),$$

(5) $$C_{t}=F_{t}\times C_{t-1}+I_{t}\times \tilde{C_{t}},$$

(6) $$h_{t}=O_{t}\times tanh\left(C_{t}\right),$$

where $I_{t}$, $F_{t}$, and $O_{t}$, correspond to the outputs of the input gate, forget gate, and output gate respectively, which are process variables used to calculate the final output; ${\tilde{C}}_{t}$ is the candidate cell state; $C_{t}$ is the cell state at time step *t*; $C_{t}$ updates the information stored in the cell state at the current step through the cell state $C_{t-1}$ at the previous step, the candidate cell state ${\tilde{C}}_{t}$ at the current step, and the gated outputs $I_{t}$ and $F_{t}$ at the current step; $x_{t}$ is the value of the input sequence at time step *t*; $h_{t}$ is the hidden state at time step *t*, which can represent all the information of the entire sequence and is calculated by the output gate result $O_{t}$ and cell state $C_{t}$ at the current step; $W_{t}$, $W_{f}$,$W_{o}$, and $W_{c}$ are the weights matrix; $\xi_{i}$, $\xi_{f}$, $\xi_{o}$, $\xi_{c}$ are the biases; σ(·) represents the sigmoid function; and tanh(·) represents the hyperbolic tangent function.

### 2.3. Encoder–Decoder Network

Encoder–decoder networks were originally employed in the translation of text or answering of language questions. Subsequently, scholars applied the LSTM architecture to the prediction of time series data, achieving favourable outcomes. The encoder–decoder network proposed by Kyunghyun Cho et al. [35] and Sutskever et al. [36], which they called the sequence2sequence model, contains two independent RNNs called the encoder and decoder. The encoder extracts the input sequence features and encodes them into a context vector *C*, which is then used as the initial hidden state input of the decoder and combined with the input time series data to obtain a new output sequence of the decoder. This process is referred to as the decoding–encoding process. In an encoder–decoder network, the context vector *C* produced by the encoder can assist the decoder in extracting time features between time series data to a greater extent, thereby enabling the decoder to achieve enhanced performance in time series data prediction tasks. However, although encoder–decoder networks are more effective at time series data prediction than a single RNN, they exhibit certain limitations. For instance, if the input time series data are of considerable length, then the input sequence may be forgotten, resulting in inadequate acquisition of the long-term characteristics of the data. The encoder context vector *C* derived in this manner is unable to fully reflect the overall characteristics of the entire long-term data series. In order to address the issue of long-term series, an attention mechanism is typically employed in the encoding–decoding process. The attention mechanism combines the context vector *C* obtained by the encoder with the input sequence of the decoder, recomputes an attention output as the input of the decoder according to different weights; it then uses the decoder to obtain a new prediction result. The advantage of this approach is that different weights can be assigned according to the relative importance of different data features at different times. Furthermore, weighting processing allows for a more accurate understanding of the overall time series data dependency of long time series, which in turn enables the generation of more accurate output results.

### 2.4. Focused Linear Attention

From the perspectives of both computational power and feature extraction, Han et al. [37] used a simple and efficient mapping function and an effective feature extraction module to introduce an efficient replacement for the self-attention mechanism called the focused linear attention mechanism. The focused linear attention mechanism not only reduces computational complexity from $O(N^{2})$ to O(N), but also has efficient feature extraction capabilities. In both the self-attention mechanism and the focused linear attention mechanism, three weight matrices are defined to compute the dependencies between the elements. These matrices are referred to as the query matrix, key matrix, and value matrix, are referred to as *Q*, *K*, and *V*. The SoftMax attentional similarity in self-attention is calculated as follows [38]:

(7) $$Sim\left(Q,K\right)=e^{\left(QK^{T}/\sqrt{d}\right)}$$

where Sim(·,·) is the formula for calculating the similarity, the calculation order is $(QK^{T})V$, and the calculation complexity is $O(N^{2})$. In the focused linear attention mechanism, *Q*, *K*, and *V* are similarly used to obtain the dependency relationship between each element. Unlike the self-attention mechanism, the similarity calculation method in the focused linear attention mechanism is as follows:

(8) $$Sim\left(Q,K\right)=\theta \left(Q\right)\theta {\left(K\right)}^{T}$$

where the function θ(x)=f(ReLU(x)), $f\left(x\right)=\frac{∥x∥}{∥x^{p}∥}x^{p}$. Subsequently, the self-attention mechanism in Equation (9) can be rewritten using the similarity calculation method of the linear attention mechanism, resulting in the expression in Equation (10):

(9) $$A_{i}=\sum_{j=1}^{n}\frac{e^{\left(Q_{i}K_{j}^{T}/\sqrt{d}\right)}}{\sum_{j=1}^{n}e^{\left(Q_{i}K_{j}^{T}/\sqrt{d}\right)}}V_{j},$$

(10) $$A_{i}=\sum_{j=1}^{n}\frac{\theta \left(Q_{i}\right)\theta {\left(K_{j}\right)}^{T}}{\sum_{j=1}^{n}\theta \left(Q_{i}\right)\theta {\left(K_{j}\right)}^{T}}V_{j}.$$

According to the associative law of matrix multiplication, the calculation order is converted from $(QK^{T})V$ to $Q(K^{T}V)$, which can be obtained as follows:

(11) $$A_{i}=\frac{\theta \left(Q_{i}\right)\left(\sum_{j=1}^{n}\theta {\left(K_{j}\right)}^{T}V_{j}\right)}{\theta \left(Q_{i}\right)\left(\sum_{j=1}^{n}\theta {\left(K_{j}\right)}^{T}\right)},$$

reducing the computational complexity of the converted data from $O(N^{2})$ to O(N). While this result represents a reduction in computational complexity, it also entails a loss of the ability to extract the features containing the most information.

In order to solve the problem of insufficient feature extraction with the linear attention mechanism, a depth-wise convolution module (DWC) is added to the focused linear attention calculation matrix, which is used to calculate several local features adjacent to each query vector in order to ensure the diversity of the overall features of the output. The output of the overall focused linear attention mechanism can be expressed as follows:

(12) $$A=Sim\left(Q,K\right)V=\theta \left(Q\right)\theta {\left(K\right)}^{T}V+DWC\left(V\right).$$

The focused linear attention mechanism offers two key advantages. First, it reduces the computational complexity of the model. Second, it has a higher feature extraction capability for data. However, the focused linear attention mechanism has a tendency to focus excessively on one aspect of the feature extraction process when applied to time series data prediction. We propose an improved version of the focused linear attention mechanism. This new mechanism allow features to be extracted from time series data in multiple subspaces. In addition, it can fully consider the data features in different subspaces and more fully understand the feature relationships in long-term time series data.

## 3. Proposed Short-Term Wind Power Composite Prediction Model: MLL-MPFLA

### 3.1. Overall Architecture

**Figure 1:**
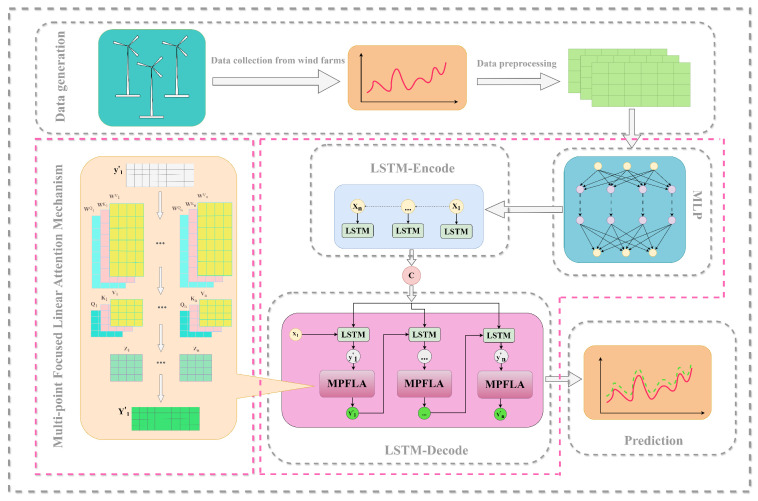
Framework of the composite MLL-MPFLA model for short-term wind power prediction.

LSTM networks have demonstrated excellent results in predicting time series data. However, their performance in multi-step prediction of multivariate time series data is unsatisfactory. Therefore, in order to enhance the accuracy of multidimensional and multi-step prediction, an MLP is employed to perform preliminary multidimensional feature extraction on the input time series data. MLP does not require convolutional computation and can process data quickly; therefore, it can be used to quickly extract multidimensional features from an input sequence. Then, a layer of the LSTM network acts as an encoder to extract the temporal correlation features and encode them to obtain the context vector *C* of the input sequence. Subsequently, another layer of the LSTM network is employed as a decoder to decode the context vector *C*. This is done in order to analyse and predict the input sequence based on the multidimensional features and temporal features stored in the context vector *C*. In the decoding process, a multi-point focused linear attention mechanism is utilised. This is done with the intention of fully considering the different features of the input sequence in the multivariate dimension and time dimension in multiple different subspaces. By calculating the multidimensional features and temporal features in multiple subspaces, a more comprehensive and accurate understanding of the feature relationship between time series data can be obtained. The prediction results obtained in different subspaces are weighted to improve the accuracy of the prediction output, and the final result is output. Figure 1 illustrates the overall MLL-MPFLA model structure.

### 3.2. Multidimensional Feature Extraction Based on MLP

An MLP is a basic neural network model that consists of one or more fully connected layers in which each neuron layer is connected to all neurons in the previous layer. MLP models are typically employed to address classification and regression problems. In addition to these tasks, they can also be utilized for data feature extraction. In this study, we consider the relationship between wind speed, temperature, pressure, and other multivariate factors influencing wind power. Long-term wind power data are initially segmented into sequences of fixed length and subsequently subjected to an MLP comprising two hidden layers for extraction of the multidimensional features. The data following the input layer are processed by linear transformation and a ReLU(·) activation function, then transmitted to the first hidden layer. To prevent overfitting, the output result is subjected to dropout processing after linear transformation in the first hidden layer prior to transmission to the subsequent layer. The second hidden layer combines the output of the first hidden layer with the original data; after linear transformation, the result is transmitted to the output layer as the output of the second hidden layer. The final data after feature extraction are obtained by linear transformation in the output layer.

### 3.3. Encoder–Decoder Network Based on LSTM

Since Kyunghyun Cho et al. [35] first proposed the encoder–decoder network model, it has gained considerable popularity among scholars engaged in the field of natural language processing. In this paper, we apply the model to the task of time series data prediction and compare it with traditional prediction models such as LSTM and MLP. Our results demonstrate that the encoder-decoder, which is typically composed of two recurrent neural networks, provides significantly enhanced prediction accuracy. In this paper, an LSTM network is employed as the encoder and decoder in light of its proven efficacy in extracting temporal features from time series data. In the encoder, the temporal features of the input sequence are extracted by the LSTM network and converted into a vector representation of fixed dimension. This conversion process is designed to retain the time correlation characteristics between the data in the entire sequence to the greatest extent possible. The specific conversion methodology is outlined below. For the sake of simplicity, we assume that the input time series data are represented by $x=[x_{1},\;x_{2},\;\ldots ,\;x_{n}]$, where $x_{t}$ represents the input data at time step *t*. At time step *t*, the LSTM network converts the input data $x_{t}$ and context vector $C_{t-1}$ of the previous step into the context vector $C_{t}$ of the current step. This conversion is represented by the function f(·):

(13) $$C_{t}=f\left(x_{t},C_{t-1}\right).$$

Consequently, the input time series data $x=[x_{1},\;x_{2},\;\ldots ,\;x_{n}]$ can be passed through the encoder to obtain a context vector *C* containing the temporal features and multidimensional feature information of the entire input sequence. The hidden state $h_{t}$ of the decoder at time step *t* is the output $y_{t-1}$ of the decoder at the previous step and the context vector *C* of the encoder as input. These are combined with the hidden state $h_{t-1}$ of the decoder at the previous step to obtain the hidden state $h_{t}$ at the current step. The function g(·) represents the transformation of the decoder’s hidden state:

(14) $$h_{t}=g\left(y_{t-1},C,h_{t-1}\right).$$

**Figure 2:**
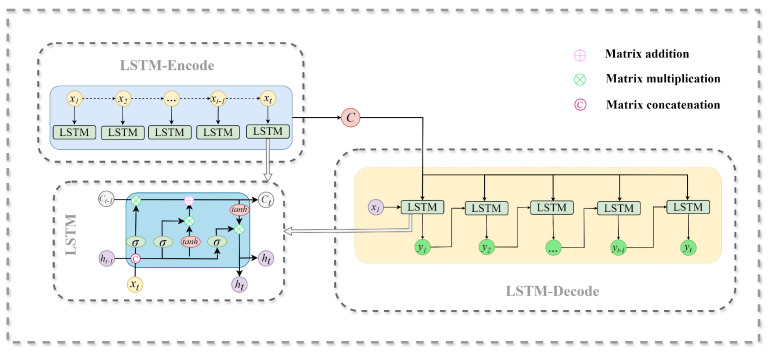
Detailed process of the LSTM-based encoder–decoder network in the proposed MLL-MPFLA model.

After obtaining the hidden state $h_{t}$ of the decoder at time step *t*, the probability output of the output $y_{t}$ at the current step is calculated by combining the output $y_{t-1}$ at the previous step. As the encoding–decoding operation delves deeper into the temporal dependency relationship between time series data, a greater number of temporal features that influence the probability output are taken into account during the calculation process. This results in more accurate prediction outcomes than those of a single LSTM network. Figure 2 illustrates the encoder–decoder network based on LSTM network.

### 3.4. Multi-Point Focused Linear Attention Mechanism

It is typical to incorporate an attention mechanism into the encoder–decoder network. This mechanism combines the hidden states of the two time series data inputs in the encoder and decoder, thereby facilitating more comprehensive feature extraction. Nevertheless, the prediction model based on the attention mechanism requires further enhancement in terms of prediction accuracy. With the objective of further improving the prediction accuracy, in this paper we employ a multi-point focused linear attention mechanism. The focused linear attention mechanism is improved by combining the characteristics of time series data; we call the resulting improved attention mechanism the multi-point focused linear attention mechanism. In the focused linear attention mechanism, the SoftMax similarity calculation method is not used; instead, the linear similarity calculation method is adopted. Although this reduces the computational complexity, it has the disadvantage of insufficient feature extraction from the data. To address this issue, the focused linear attention mechanism employs a deep convolution module to convolve and extract multiple adjacent features in close proximity to each *V*, thereby extracting more data features [37]. This process is described by Equation (12). Because the focused linear attention mechanism performs convolutional feature extraction on *V*, and because *V* is obtained through linear calculation, part of the original information contained in the time series data is lost, resulting in incomplete feature extraction from the time series data. Taking this into account, we perform convolutional feature extraction directly on the input data of each time step in the multi-point focused linear attention mechanism, replacing the deep convolution module in the focused linear attention mechanism. We use CONV(x) to represent the convolutional feature extraction operation on the input sequence of each time step, which we use to replace the DWC(V) module in Equation (12). Then, the output of the improved focused linear attention mechanism can be described by Equation (15):

(15) $$A=Sim\left(Q,K\right)V=\theta \left(Q\right)\theta {\left(K\right)}^{T}V+CONV\left(x\right).$$

The advantage of this approach is that it can fully consider the characteristics of the time series data and use the original data for feature extraction directly, reducing the loss of features to ensure that more complete features are extracted from the input time series data sequence.

**Figure 3:**
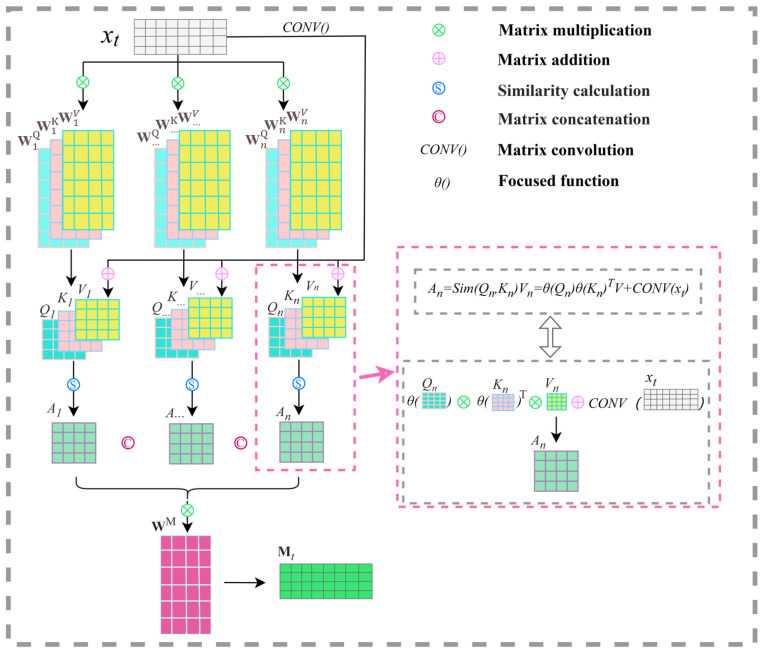
Detailed process of the multi-point focused linear attention mechanism in the proposed MLL-MPFLA model.

During calculation, the focused linear attention mechanism may focus unduly on the features in a certain subspace while ignoring the feature information of other subspaces. To address this issue, the multi-point focused linear attention mechanism proposed in this paper employs a strategy that fully leverages the feature information across multiple subspaces. This involves initializing the focused linear attention mechanism into multiple groups of distinct *Q*, *K*, and *V*, calculating the attention output corresponding to each group of *Q*, *K*, and *V*, then weighting multiple different attention outputs to obtain a new attention output. As shown in Figure 3, the data $x_{t}$ at the current time step are matrix-multiplied with multiple sets of different projection matrices to obtain multiple sets of different *Q*, *K*, and *V*. Equation (16) describes this process:

(16) $$Q_{n}=x_{t}W_{n}^{Q},\;K_{n}=x_{t}W_{n}^{K},\;V_{n}=x_{t}W_{n}^{V}$$

where $Q_{n}$, $K_{n}$, and $V_{n}$ respectively represent the *Q*, *K*, and *V* of the *n*th subspace at time step *t*, $x_{t}\in R^{N\times C}$ represents the input data at time step *t*, and $W_{n}^{Q},\;W_{n}^{K},\;W_{n}^{V}\in R^{C\times C}$ are projection matrices. Then, the corresponding attention outputs are calculated based on the multiple sets of *Q*, *K*, and *V*. Equation (17) describes this process:

(17) $$A_{n}=\theta \left(Q_{n}\right)\theta {\left(K_{n}\right)}^{T}V_{n}+CONV\left(x_{t}\right)$$

where $Q_{n}$, $K_{n}$, and $V_{n}$ respectively represent the *Q*, *K*, and *V* of the *n*th subspace at time step *t*, while $A_{n}$ represents the attention output of the *n*th subspace at time step *t*. Finally, we concatenate the multiple sets of attention outputs and multiply them by a projection matrix to obtain the final attention output. Equation (18) describes this process:

(18) $$M_{t}=Concat\left(A_{1},\;\ldots ,\;A_{n}\right)W^{M}$$

where $M_{t}$ represents the multi-point focused attention output at time step *t*, Concat(·) represents matrix concatenation, and $W^{m}\in R^{nC\times C}$ is the projection matrix.

After calculating the attention output of the multi-point focused linear attention mechanism, this attention output can be used to more accurately analyze the input time series data during the decoding process, thereby obtaining better prediction results. The multi-point focused linear attention mechanism calculates multiple sets of different initial values *Q*, *K*, and *V* in the same improved focused linear attention mechanism to fully consider different features in multiple subspaces. Compared with the focused linear attention mechanism, it can capture more relational features of time series data in multiple different subspaces, thereby further improving the accuracy of time series data prediction results.

## 4. Experiment and Analysis

In this section, we first provide a description of the real dataset and preprocessing used in our experiments. Then, we describe the experimental verification conducted on this dataset, with five other prediction methods commonly used as benchmark models included for comparison with the proposed composite prediction model. The effectiveness of the proposed MLL-MPFLA model is proven by comparing these models on several performance indicators.

### 4.1. Experimental Data and Preprocessing

**Figure 4:**
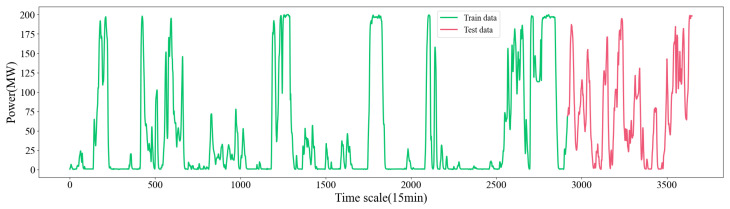
Actual wind power data in the dataset.

The dataset utilised in this study is described in this subsection. This dataset is derived from the actual wind power generation data of a power plant in Xinjiang, China, as documented in the Aliyun Tianchi dataset. The dataset contains 3649 samples collected every 15 min. Each sample includes eleven environmental influencing factors along with actual power generation data. The eleven influencing factors include the wind speed at 10 m, 30 m, 50 m, and 70 m away from the power generation equipment, the wind direction at 10 m, 30 m, 50 m, and 70 m away from the power generation equipment, and the temperature, air pressure, and humidity near the power generation equipment at the current moment. Because the impact of wind direction data on power generation is not highly correlated, in this study only the impacts of wind speed, temperature, air pressure, and humidity at 10 m, 30 m, 50 m, and 70 m away from the power generation equipment on the actual power generation are considered. Selected data from the dataset are shown in Table 1. In this study, the total samples are divided into two parts, as illustrated in Figure 4; the first 80% of the samples are designated as training samples, while the remaining 20% constitute test samples. Table 2 provides a statistical description of the dataset.

For data preprocessing, considering that wind power data are discrete, the data were smoothed first. The advantage of this approach is that it can reduce the noise interference in the original data, eliminate the impact of random fluctuations, and enable the neural network model to better analyze and process the data. In this paper, KalmanFilter smoothing was selected; other methods for smoothing include exponential smoothing, polynomial smoothing, Gaussian smoothing, and more. Then, we used Z-Score standardization to convert the data to a unified scale. After Z-Score standardization, the mean of the data was adjusted to 0 and the standard deviation was adjusted to 1. Finally, we divided the entire data set into multiple segments using a sliding window of size 20. In each segment, the first 16 datapoints are used as the batch size for model training and the last four are used as labels to verify the prediction results. Therefore, the MLL-MPFLA model can use the wind power generation data of the past four hours (i.e., sixteen wind power generation data points) to predict the wind power generation in the next hour (i.e., the next four moments). Because the data in this dataset are highly complete with no missing data, we did not perform any missing data processing. The data used in all experiments described in this article are based on the above preprocessing approach.

### 4.2. Evaluation Metrics

In order to evaluate the accuracy of the MLL-MPFLA model in wind power generation prediction, three commonly used quantitative indicators are used as performance evaluation indicators: mean absolute error (MAE), root mean square error (RMSE), mean absolute percentage error (MAPE), and coefficient of determination ($R^{2}$). These can be respectively expressed by the following formulas:

(19) $$\mathrm{MAE}=\frac{1}{n}\sum_{i=1}^{n}\left|\tilde{P_{i}}-T_{i}\right|,$$

(20) $$\mathrm{RMSE}=\sqrt{\frac{1}{n}\sum_{i=1}^{n}{\left(\tilde{P_{i}}-T_{i}\right)}^{2}},$$

(21) $$\mathrm{MAPE}=\frac{1}{n}\sum_{i=1}^{n}\left|\frac{T_{i}-\tilde{P_{i}}}{T_{i}}\right|\times 100\%,$$

(22) $$R^{2}=1-\frac{\sum_{i=1}^{n}{\left(T_{i}-\tilde{P_{i}}\right)}^{2}}{\sum_{i=1}^{n}{\left(T_{i}-\bar{T}\left(i\right)\right)}^{2}},$$

where $\tilde{P_{i}}$ is the predicted value, $T_{i}$ is the true value, and $\bar{T}\left(i\right)$ is the mean of the actual values.

### 4.3. Analysis of Wind Power Generation Prediction Results

**Figure 5:**
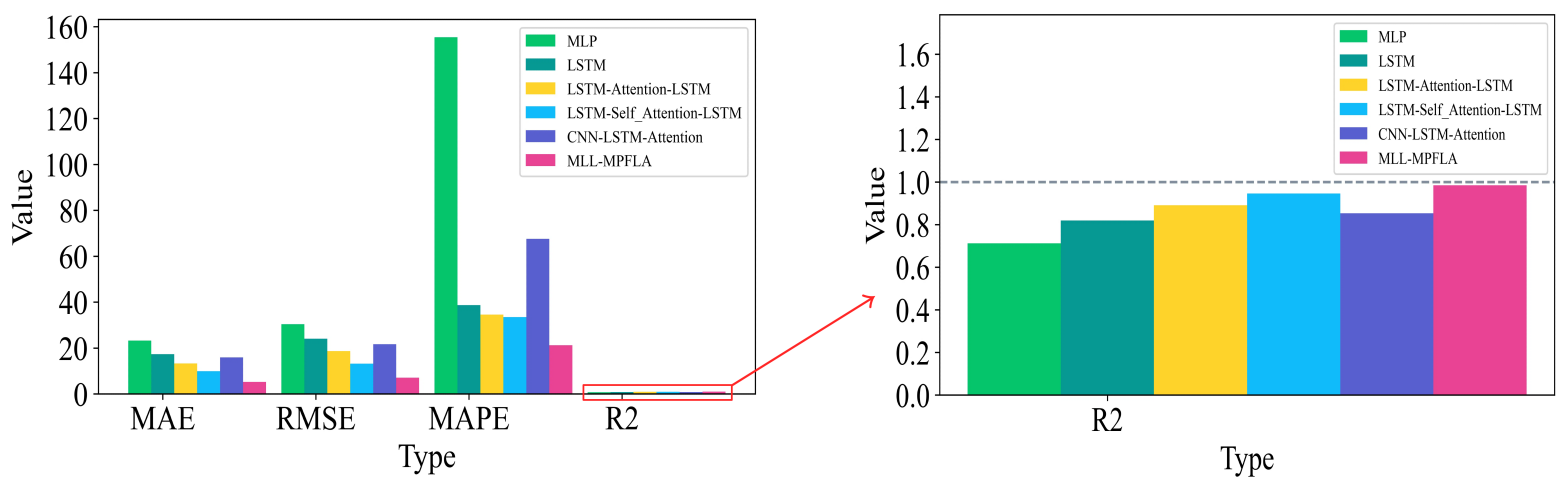
Comparison of MLL-MPFLA evaluation metrics with the five benchmark models.

**Figure 6:**
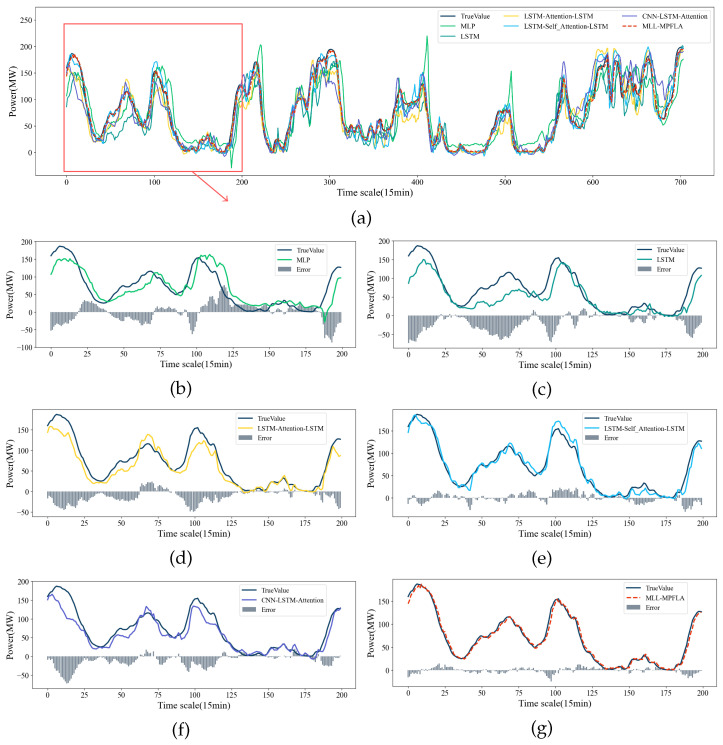
Short-term wind power prediction results for the five different methods: (**a**) shows all predicted results, (**b**–**f**) show the partial prediction results of the five benchmark models, and (**g**) shows the MLL-MPFLA partial prediction results.

In order to assess the efficacy of the proposed MLL-MPFLA model, five commonly used prediction models were selected as benchmark models for comparative experiments. The specific settings of the benchmark models are presented in Table 3. The LSTM–Attention–LSTM and CNN–LSTM–Attention models were proposed in [18,21], respectively. In this paper, we conducted cross-validation through a large number of experiments and select the best hyperparameters in the MLL-MPFLA model based on empirical settings. The specific hyperparameter settings are shown in Table 4, taking the number of hidden units in the LSTM network’s decoder and encoder as an example. We first set the initial value of the number of units to 8 and increased the number of units by multiples of 8 each time until the best parameter setting was obtained. We verified whether the parameters were optimal by comparing the MAE and RMSE indicators. The encoder and decoder in the MLL-MPFLA model used the same LSTM hyperparameter settings, with 3 hidden layers, 512 hidden units, 0.001 learning rate, 0.05 dropout, and 260 training epochs. The convolution layer parameters for convolution feature extraction of the input time series were set as follows: the number of channels was set to 16, the number of convolution kernels to 16, the convolution kernel size to 1×1, and the stride of the convolution to 1. In the MLP used for multidimensional feature extraction, the number of units in the first hidden layer was set in the same way as in the above method; the specific number of hidden layers units was set to 512. In order to ensure the consistency of the output data dimension, the number of units in the second hidden layer was set to 8. Following [29], we used two hidden layers and a dropout value of 0.1. In the encoding–decoding process, we utilised the multi-point focused linear attention mechanism in 8 subspaces for weighted sum prediction. The hyperparameter setting method of the benchmark models was the same as for the MLL-MPFLA model: the number of MLP hidden layers was set to 3, the number of hidden layers units in the first and second hidden layers to 512, the number of hidden layer units in the third hidden layer to 8, the dropout to 0.1, the activation function was ReLU(·), and the number of training rounds was 10. The number of hidden layers of the LSTM was set to 3, the number of hidden layer units to 512, the learning rate to 0.001, the dropout to 0.05, and the training round to 150. In LSTM–Attention–LSTM, the LSTM hyperparameter settings used by the encoder and decoder were the same: the number of hidden layers was set to 3, the number of hidden layer units to 512, the learning rate to 0.001, the dropout to 0.05, and the number of training epochs to 260. In LSTM–Self_Attention–LSTM, the LSTM hyperparameter settings used by the encoder and decoder were the same: the number of hidden layers was set to 3, the number of hidden layer units to 512, the learning rate to 0.001, the dropout to 0.05, and the number of training epochs to 260. In the CNN–LSTM–Attention model, the number of LSTM hidden layers was set to 2, the number of hidden layer units to 256, the learning rate to 0.001, and the dropout to 0.05. For the CNN, the number of channels was set to 256, the number of convolution kernels to 4, and the number of training epochs to 100. All of the above LSTM networks were implemented using the LSTM class in Pytorch 2.2.2. Considering that the prediction results of neural networks are random, multiple experiments were conducted in order to reduce random errors, taking the average of the results. We conducted five repetitions, with the experimental results shown in Table 3 and Figure 5. The prediction results are shown in Figure 6a. Furthermore, all of the aforementioned models were executed on a server equipped with a 3.5 GHz Intel Core i7-13700K processor, an NVIDIA GeForce RTX 4090 graphics processing unit (GPU), and 32 GB of memory, as illustrated in Table 5.

#### 4.3.1. Performance Analysis of the Models

Table 3 and Figure 5 demonstrate that the MLL-MPFLA model proposed in this paper outperforms the five benchmark models in short-term wind power prediction. Figure 5 shows intuitively that the MLL-MPFLA model has the lowest MAE, RMSE, and MAPE indicators along with the highest $R^{2}$ indicator. From Table 3, the proposed model’s MAE is the lowest at 5.2124, its RMSE is the lowest at 7.0972, and its $R^{2}$ is the highest at 0.9843. The LSTM–Self_Attention–LSTM model is the second-best performer, with an MAE value of 9.9060, RMSE value of 13.1949, and $R^{2}$ value of 0.9457. The MLP model is the least effective, with MAE, RMSE, and $R^{2}$ values of 23.2081, 30.4275, and 0.7119, respectively. The $R^{2}$ index is a measure of the degree of fit between the prediction result and the true value, with higher values indicating a greater degree of fit. As illustrated in Table 3, the $R^{2}$ index of the proposed MLL-MPFLA model exhibits a notable increase relative to the benchmark models, reaching 0.2724, 0.1646, 0.0931, 0.0386, and 0.1310, respectively. This indicates that the MLL-MPFLA model exhibits the most optimal fit. The MAE and RMSE results for MLP are 23.2081 and 30.4275, respectively. Compared with MLP, the MAE and RMSE of the proposed model are respectively reduced by 17.9957 and 23.3303. The superiority of MLL-MPFLA over MLP lies in the extraction and analysis of the temporal characteristics of wind power data and the use of the multi-point focused linear attention mechanism to fully consider the impact of temporal characteristics on power generation. From the analysis of the MAPE index, in Figure 6a, it can be seen that the MAPE index of MLP is high because the prediction error for certain data points is large when MLP predicts data close to 0; thus, no comparison analysis with the MAPE index of the MLL-MPFLA model is possible. Similarly, the MAE, RMSE, and MAPE of LSTM are 17.3552, 24.0803, and 38.7232% respectively. Compared with LSTM, the MAE, RMSE, and MAPE of the MLL-MPFLA model are reduced by 12.1428, 16.9831, and 17.4987%, respectively. The superiority of MLL-MPFLA over the baseline LSTM network lies in its deeper extraction and analysis of the multidimensional characteristics of wind power data and the use of an encoder–decoder network based on LSTM, which enhances the LSTM network’s ability to analyze the temporal characteristics of data. Compared with LSTM–Attention–LSTM, the MAE, RMSE, and MAPE of the proposed model are reduced by 8.0202, 11.5786, and 13.3184%, respectively. Compared with LSTM–Self_Attention–LSTM, the MAE, RMSE, and MAPE of the proposed model are reduced by 4.6936, 6.0977, and 12.2826%, respectively. The superiority of MLL-MPFLA over these two comparative models lies in its deeper extraction and analysis of the multidimensional features of wind power data and its use of a more efficient multi-point focused linear attention mechanism to fuse the multidimensional time series data features in multiple subspaces and fully extract the features of the time series data, thereby obtaining better prediction performance. In addition, compared with CNN–LSTM–Attention, the MAE, RMSE, and MAPE of the proposed model are reduced by 10.7471, 14.5932, and 46.3280% respectively. This is because the proposed model uses a special encoding–decoding operation to enhance the feature extraction capability for time series data. In addition, the proposed model uses a multi-point focused linear attention mechanism with stronger feature extraction capability, which is an indispensable factor in its achieving better prediction results. Through the above analysis, we can draw the following conclusions. The MLL-MPFLA model proposed in this paper represents the optimal performance. It analyses and combines the multidimensional and temporal features of time series data, then weights the prediction results of different dimensions through the multi-point focused linear attention mechanism, thereby obtaining superior prediction performance.

#### 4.3.2. Error Analysis of Model Prediction Results

**Figure 7:**
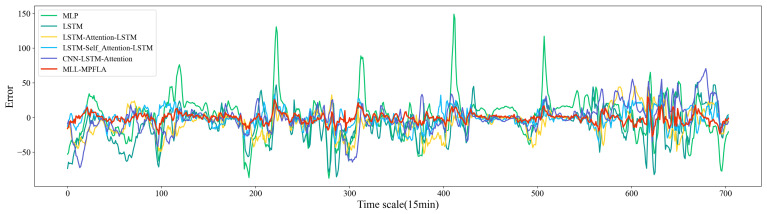
Error comparison between MLL-MPFLA and the five benchmark models.

The error of each model is shown in Figure 7, where the error calculation $Error=P_{i}-T_{i}$, $P_{i}$ represents the predicted value and $T_{i}$ represents the true value. It can be observed that the error of the proposed model is smaller than that of other models, indicating that the accuracy of the prediction results is relatively high. In theory, it is desirable for the difference between the predicted value and the true value to be infinitely close to 0; however, from the actual prediction results it can be seen that this is difficult to achieve. In actual prediction tasks, a smaller difference between the predicted value and true value indicates a better prediction effect. The red curve in Figure 7 represents the prediction error of the MLL-MPFLA model, exhibiting a floating range near 0. Overall, the prediction error is smaller than that of the five compared benchmark models. For the shallow MLP neural network, only the impact of multiple environmental factors on the power generation is considered, without considering the impact of time series characteristics on power generation; thus, the prediction result has a large error. The MLL-MPFLA model fully extracts and analyzes the multidimensional characteristics and time series characteristics of wind power data at the same time, meaning that the prediction error is greatly reduced compared with the MLP. For the LSTM network, although it can extract and analyse the time series characteristics of wind power data, its ability to extract multidimensional features of wind power data is obviously insufficient compared with the MLL-MPFLA model, resulting in a higher prediction error. For the LSTM-based encoder–decoder network, the time characteristics of the data can be extracted and analyzed; although the prediction error is significantly reduced compared to MLP and LSTM, it is still higher than our proposed MLL-MPFLA model. This is because the MLL-MPFLA model not only designs a separate multidimensional feature extraction module for wind power data but also uses a superior multi-point focused linear attention mechanism, resulting in the prediction error of the MLL-MPFLA model being lower than that of the LSTM-based encoder–decoder network. In addition, although the prediction errors shown in Figure 7 are smaller at some moments than those of the MLL-MPFLA model, the overall proportion of these points with smaller errors is very small. This phenomenon is due to the random nature of the neural network model’s prediction output, which results in the appearance of points that are closer to the true power value, thereby reducing the error compared to the MLL-MPFLA model. With the exception of a few points that may be attributed to randomness, the overall error analysis indicates that the prediction accuracy of the MLL-MPFLA model is superior to that of the other models.

#### 4.3.3. Effectiveness Analysis of Model Prediction Results

A comparison of the prediction results with those of the other five benchmark models is presented in Figure 6. Figure 6a shows the comparison of the prediction results of all models, while Figure 6b–f shows local enlarged prediction diagrams of the five benchmark models. Figure 6g is a local enlarged prediction diagram of the proposed MLL-MPFLA model. The bars in the local enlarged diagram represent the error size of the current point. The figure illustrates that the prediction result curve of the proposed model exhibits the highest degree of fit with the true value curve accompanied by the smallest error, indicating the most accurate prediction results. In addition, it can be seen from Figure 6b that the prediction error of MLP near the value of 0 is large; in particular, when the data fluctuate greatly near the value, the prediction effect is the worst. This situation causes the MAPE index to soar, making the MAPE index of MLP higher than that of MLL-MPFLA. The reason for this phenomenon is that the shallow neural network MLP does not extract the time features of the time series data and its extraction of multidimensional features is not sufficient, resulting in the worst prediction performance and the most obvious decline in fit compared with the MLL-MPFLA model. The LSTM network demonstrates commendable performance in the time series data prediction task; however, numerous factors have an impact on the prediction outcomes of wind power data in this study. The single LSTM network has a limited effect on multivariate feature extraction, and its prediction effect is significantly inferior to that of the MLL-MPFLA model. In comparison to the LSTM network, the MLL-MPFLA model has a distinct network module for deep extraction of the multidimensional features of the time series data. Additionally, it employs a multi-point focused attention mechanism to assign varying weights to the prediction results. Through continuous learning and training, the optimal weight matrix can be identified, enabling the generation of optimal prediction results. While the prediction efficacy of the LSTM-based encoder–decoder network is considerably superior to that of the shallow MLP neural network, its fit remains inferior to that of the MLL-MPFLA model. This is primarily reflected in the substantial discrepancy in prediction outcomes when the data exhibit significant fluctuations. This phenomenon is due to the model’s incomplete learning of the multidimensional features that influence wind power data, which results in suboptimal prediction outcomes when the data exhibit significant fluctuations. From the fit analysis of the prediction results of each model, it can be seen that the proposed MLL-MPFLA model fully considers the impact of multidimensional features and temporal features on the prediction results, uses a more efficient multi-point focused linear attention mechanism, and obtains the best prediction results compared with the other five benchmark models.

### 4.4. Generalization Experiment

**Figure 8:**
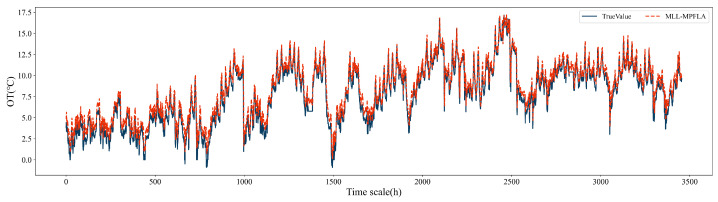
Prediction results using MLL-MPFLA on the ETTh1 dataset.

Without readjusting the hyperparameters of the proposed model, the generalization of the model was verified using the public ETTh1 dataset [39]. The experimental results are shown in Figure 8. It can be seen from the figure that the prediction results of the MLL-MPFLA model are highly consistent with the real data. Similarly, without readjusting the hyperparameters of the benchmark model, the other five benchmark models were used on the same dataset. The $R^{2}$ indexes of MLP, LSTM, LSTM–Attention–LSTM, LSTM–Self_Attention–LSTM, CNN–LSTM–Attention, and MLL-MPFLA are 0.6464, 0.8042, 0.7436, 0.7860, 0.6890, and 0.9145 respectively. From the $R^{2}$ index, it can be seen that the MLL-MPFLA model has the highest degree of fit on different datasets.

In conclusion, the MLL-MPFLA model proposed in this paper demonstrates a notable enhancement in comparison to other benchmark models across the three dimensions of performance indicators, result errors, and prediction result fitting effects. This evidence substantiates the effectiveness and reliability of the proposed model in wind power data prediction and validates its potential as a robust analytical and predictive tool for power grid security maintenance. The prediction time of all methods was statistically analyzed under the server configuration shown in Table 5. The results show that the prediction time required by all models is less than 0.2 s for the test data (size 4×16×8 bytes), which can meet the needs of most real environments, including resource-constrained environments. However, in the MLL-MPFLA model, because the hyperparameters were empirically set through a large number of experiments, the hyperparameters need to be reset when the dataset changes. In addition, as with most prediction models, the prediction effect of our proposed model will tend to decline when the prediction step size increases.

## 5. Conclusions

The prediction of wind power generation represents an effective measure for the stable operation of power grids. The superiority of the MLL-MPFLA model proposed in this paper is evident in its ability to separately extract multidimensional features and temporal features of time series data while fully considering the correlation between the two. Furthermore, a more efficient multi-point aggregation linear attention mechanism is employed to fully consider the varying importance of different features from multiple subspaces, enabling more accurate predictions. The following is a summary of the full text. First, an MLP is employed to extract the multidimensional features of a multitude of factors that influence power generation. Subsequently, the multidimensional features and temporal features are integrated and predicted in conjunction with the LSTM-based encoder–decoder network model. The advantage of this approach is that the time series data can be fully mined and associated in both the multivariate dimension and the time correlation dimension. In the decoding process, the multi-point focused linear attention mechanism is used to weight the different features of the wind power data in multiple subspaces. This approach fully considers the distinct features present in each subspace and integrates features across multiple dimensions, thereby enhancing the accuracy of the prediction. A case study of a wind power dataset from Xinjiang, China was conducted to compare the MLL-MPFLA model with five benchmark models: MLP, LSTM, LSTM–Attention–LSTM, LSTM–Self_Attention–LSTM, and CNN–LSTM–Attention. The efficacy of the MLL-MPFLA model was then demonstrated through a comparative analysis of four evaluation metrics (i.e., MAE, RMSE, MAPE, and $R^{2}$), an error analysis of the prediction results, and an effect analysis of the prediction curves. In summary, the MLL-MPFLA model proposed in this paper can make accurate predictions of future short-term power generation based on wind power data generated in a previous period of time. It can then make correct responses in advance according to the prediction results, ensuring the safe maintenance of the power grid and reducing the occurrence of accidents. Because the hyperparameters of our model are empirically set through experiments, in subsequent work optimization methods such as Bayesian optimization could be used to reduce the workload of empirical hyperparameter setting by automatically optimizing the hyperparameters of the model. In addition, it would be possible to introduce the attention mechanism into the extraction of multidimensional features and improve the ability of the model to extract multidimensional features of data through the attention mechanism, allowing it to achieve higher prediction accuracy while enhancing its ability to predict data at more unknown time points in the future.

## Figures and Tables

**Table 1:** Selected data from the experimental dataset.

Time (1 January 2019)	10 m Wind Speed (m/s)	30 m Wind Speed (m/s)	50 m Wind Speed (m/s)	70 m Wind Speed (m/s)	Temperature	Air Pressure	Humidity	Actual Generating Power (MW)
00:00	0.7985	0.8332	0.8552	0.9023	1.4492	55.6030	4.2044	0.8419
00:15	1.4496	1.4503	1.6260	1.6774	4.8586	294.4792	19.7361	1.8593
00:30	1.6911	1.8676	2.2179	2.4523	8.0877	520.9940	34.3980	2.5457
00:45	2.1097	2.1228	2.7735	3.0864	10.228	671.2503	44.2247	2.5929
01:00	2.5557	2.4614	3.0564	3.3157	11.4889	759.8624	50.2560	3.1226

**Table 2:** Statistical information of the dataset.

Dataset	Max	Median	Min	Mean	Std.
Entire_dataset	199.9478	14.6293	0.2958	49.8576	65.1104
Training_dataset	199.9478	5.5637	0.2958	43.2053	65.1744
Test_dataset	198.9684	71.2219	0.4953	76.4488	57.6370

**Table 3:** Evaluation metrics from five experiments on MLL-MPFLA and the five benchmark models.

	Item	MLP	LSTM	LSTM-Attention-LSTM	LSTM-Self_Attention-LSTM	CNN-LSTM-Attention	MLL-MPFLA
No.1	MAE	23.1331	17.5249	12.069	9.1511	14.3541	5.1588
	RMSE	30.1929	23.9201	16.6325	12.0874	20.6491	7.0523
	MAPE (%)	167.0769	44.5796	38.4315	37.1742	59.3579	19.5516
	$R^{2}$	0.7167	0.8222	0.9140	0.9546	0.8675	0.9845
No.2	MAE	22.6388	18.122	12.6918	10.2816	16.3484	5.1479
	RMSE	29.2637	24.9896	18.2958	13.8428	21.8186	6.9892
	MAPE (%)	139.7371	40.1032	35.6961	35.0988	77.6622	20.0215
	$R^{2}$	0.7338	0.8059	0.8960	0.9404	0.8520	0.9848
No.3	MAE	23.7125	17.4509	13.3007	9.2505	17.3603	5.3487
	RMSE	31.4384	24.3349	19.0554	12.3393	23.9476	7.2316
	MAPE (%)	134.9939	37.7755	32.1601	29.6697	48.4913	23.3469
	$R^{2}$	0.6928	0.8159	0.8871	0.9527	0.8218	0.9837
No.4	MAE	24.9549	17.0290	13.9663	10.4409	16.0365	5.1055
	RMSE	31.9169	23.8013	19.7611	13.9102	21.1661	7.0302
	MAPE (%)	184.4613	35.5131	32.5987	32.8792	62.8921	19.8950
	$R^{2}$	0.6834	0.8239	0.8786	0.9399	0.8608	0.9846
No.5	MAE	21.6011	16.6492	14.1351	10.4061	15.6982	5.3009
	RMSE	29.3257	23.3557	19.6341	13.795	20.8707	7.1826
	MAPE (%)	150.7046	35.6445	33.8280	32.7137	89.3589	23.3075
	$R^{2}$	0.7327	0.8305	0.8802	0.9409	0.8646	0.9840
Avg	MAE	23.2081	17.3552	13.2326	9.9060	15.9595	5.2124
	RMSE	30.4275	24.0803	18.6758	13.1949	21.6904	7.0972
	MAPE (%)	155.39476	38.7232	34.5429	33.5071	67.5525	21.2245
	$R^{2}$	0.7119	0.8197	0.8912	0.9457	0.8533	0.9843

**Table 4:** MLL-MPFLA model hyperparameter configuration.

Type	Configuration	Value
Parameters of MLP	Number of hidden layers	2
	Number of first hidden layer units	512
	Number of second hidden layer units	8
	Dropout	0.1
	Activation function	ReLU(·)
	Number of training epochs	260
Parameters of LSTM in Encoder and Decoder	Number of hidden layers	3
	Number of hidden layer units	512
	Dropout	0.05
	Learning rate	0.001
	Number of training epochs	260
Parameters of convolution in MLL-MPFLA	Number of channels	16
	Kernel number of convolution	16
	Kernel size of convolution	1×1
	Strider of convolution	1

**Table 5:** Server configuration information.

Type	Parameter
CPU	13th gen Intel(r) Core(tm) i7-13700K
GPU	NVIDIA GeForce RTX 4090
Memory	32G
Pythorch	2.2.2

## Data Availability

The experimental data used in this study come from the Alibaba Cloud Tianchi dataset https://tianchi.aliyun.com/dataset/159885 (accessed on 22 April 2024); the ETTh1 dataset used in the generalization experiment come from https://github.com/zhouhaoyi/ETDataset (accessed on 26 March 2024).
